# Impact of Individual Process Parameters on Extraction of Polysaccharides from *Saccharina latissima*

**DOI:** 10.3390/md23110435

**Published:** 2025-11-13

**Authors:** Elmira Khajavi Ahmadi, Said Al-Hamimi, Madeleine Jönsson, Roya R. R. Sardari

**Affiliations:** 1 Division of Biotechnology and Applied Microbiology, Department of Process and Life Science Engineering, Lund University, Naturvetarvägen 22, 223 62 Lund, Sweden; 2 Centre for Analysis and Synthesis, Department of Chemistry, Lund University, Naturvetarvägen 22, 223 62 Lund, Sweden; 3 Department of Food and Meal Science, Kristianstad University, Elmetorpsvägen 15, 291 39 Kristianstad, Sweden

**Keywords:** *Saccharina latissima*, conventional extraction, pressurized liquid extraction (PLE), moderate electric field (MEF), marine polysaccharides

## Abstract

While numerous extraction methods have been applied to the brown algae *Saccharina latissima*, a systematic evaluation of how individual extraction parameters influence the extraction of each target polysaccharide has not previously been reported. Accordingly, this study compared conventional and advanced techniques for extracting fucoidan, laminarin, and alginate from pre-treated biomass. Conventional methods employed diluted acid (0.01 M and 0.1 M HCl), diluted alkali (0.01 M and 0.1 M NaOH), and hot water (121 °C for 30/60 min) for extraction. Advanced techniques involved pressurized liquid extraction (PLE) using water and moderate electric field (MEF) extraction with conditions optimized by statistical experimental design. Pre-treatment with aqueous ethanol removed 30% ash and eliminated mannitol, improving extraction selectivity. The results demonstrated fucoidan yields of 31% with 0.01 M HCl and 46% with 0.1 M NaOH, while 0.01 M NaOH facilitated laminarin co-extraction (45%). Alginate, as a mannuronic acid polymer, was obtained at 9% yield with 0.1 M HCl, 42% yield with 0.1 M NaOH, and 27% with pressurized hot water for 30 min. High-temperature, short-duration PLE further improved alginate yield, while MEF showed limited gains due to high ionic content but demonstrated potential under optimized settings. The results support a cascading biorefinery approach in which different polysaccharide fractions can be sequentially obtained, contributing to more sustainable seaweed valorization.

## 1. Introduction

The marine environment, with its remarkable biodiversity, holds immense potential as a source of bioactive and chemically diverse macromolecules, attracting attention in different fields of application [1]. Seaweed (marine macroalgae) is a valuable and commercially important marine resource, long used for its rich content of nutrients and bioactive compounds such as polysaccharides, proteins, and lipids [2]. Global seaweed production continues to rise, with over 30 million tons of fresh seaweed (both cultivated and wild) harvested in 2022. Brown seaweed (*Phaeophyceae*) accounted for approximately 40-50% of this volume [3]. Brown seaweeds are gaining increasing attention as a sustainable biomass source for the production of high-value bioactive compounds. Among them, *Saccharina latissima* (L.) C.E.Lane, C.Mayes, Druehl & G.W.Saunders 2006 (sugar kelp) is one of the most commercially important species. This species is native to the cold-temperate waters of the North Atlantic and North Pacific, and is recognized in the European Union’s novel food catalogue, confirming its suitability and safety for human consumption [4].

*S. latissima* is rich in various carbohydrates and ash (minerals), predominantly sodium, potassium, calcium, magnesium, chlorine, bromine, iodine, phosphorus, and sulfur [5,6]. To fully unlock the biotechnological and commercial potential of *S. latissima*, it is essential to develop selective and efficient extraction processes that allow the isolation of these compounds with minimal degradation and cross-contamination [7].

*S. latissima* contains several valuable carbohydrates, including water-soluble polysaccharides such as fucoidan, laminarin, and the sugar alcohol mannitol, as well as water-insoluble ones such as alginate and cellulose [5]. In contrast to other algal groups, fucoidan, alginate, and cellulose represent the primary structural polysaccharides exclusively in the cell walls of *S. latissima* belonging to *Phaeophyceae*. [7]. Fucoidan is a heterogeneous sulphated fucose-rich polymer with reported antiviral and anti-inflammatory effects, and alginate, a structural polysaccharide widely utilized in the food, pharmaceutical, and biomedical sectors due to its gelling, thickening, and stabilizing capabilities. Alginates are linear copolymers made up of (1→4)-linked β-D-mannuronic acid (M) and α-L-guluronic acid (G). Their high intrinsic viscosity, excellent water-binding capacity, and ability to form hydrogels make them valuable for a wide range of commercial applications. Laminarin is located within the cell vacuoles of the fronds and is a low molecular weight biopolymer and a storage β-glucan with recognized prebiotic and antioxidant properties [7].

Current extraction strategies for seaweed polysaccharides often involve the use of pre-treatments, cell disruption techniques, as well as conventional and advanced extraction processes. Fucoidan and laminarin are typically extracted using mild to moderate acidic conditions, combined with elevated temperatures and water as solvent. Optimization of pH, temperature, and extraction time is critical to maximize yields and preserve structural integrity [8,9].

For alginate extraction, acidification is applied to convert alginate salts into insoluble alginic acid, enhancing both yield and extraction efficiency. Controlled conditions with low pH, moderate temperature, and optimized extraction time are crucial to maximize recovery while avoiding degradation. Following acid treatment, alkaline extraction is performed by adjusting the pH to 9–10, converting alginic acid to water-soluble alginate salts, with overall efficiency influenced by temperature, alkali concentration, pH, duration, and their combined effects [10].

While multiple extraction methods have been applied to *S. latissima*, the optimal conditions can vary not only between algal species but also among different tissues of the same species [10]. Therefore, a focused study on the effects of individual extraction parameters on each polysaccharide could yield important insights and deepen the understanding of their extraction dynamics.

Advanced methods for the extraction of bioactive compounds have recently emerged, driven by the need for more sustainable and eco-friendly extraction methods. Pressurized liquid extraction (PLE) has been applied to *S. latissima* for polyphenol recovery [11], but not yet for polysaccharide extraction. Electric field-based techniques such as pulsed electric field (PEF) and moderate electric fields (MEF) permeabilize cell membranes via electroporation, enhancing mass transfer and improving compound yields [12,13]. This approach has been used to extract potentially toxic elements (PTEs) like iodine from *S. latissima* but has not been explored for polysaccharides [12].

This study aimed to investigate and optimize the extraction of fucoidan, laminarin, and alginate from *S. latissima* biomass using both conventional and advanced techniques. The specific objectives were to:

- Characterize the composition of *S. latissima* biomass to establish a baseline for extraction.
- Evaluate the effectiveness of conventional extraction methods, including extractions with diluted acid (HCl), diluted alkali (NaOH), and hot water, for polysaccharide yield and composition.
- Assess the efficacy of two advanced extraction techniques, pressurized liquid extraction (PLE) and moderate electric field (MEF) extraction, for polysaccharide recovery from pre-treated seaweed biomass.
- Compare yields, compositions, selectivity, and purity across all methods.
- Isolate and analyze the crude extracts of fucoidan, laminarin, and alginate to evaluate component profiles, selectivity, and extraction efficiency.

## 2. Results

### 2.1. Characterization of Untreated and Pre-Treated S. Latissima Biomass

Table 1 presents the carbohydrate and ash composition of untreated *Saccharina latissima* biomass, while Table 2 shows the composition of the biomass following pre-treatment with aqueous ethanol solution (80% *v/v*).

A comparison of the results in Table 1 and Table 2 demonstrated that mannitol was almost completely removed during the pre-treatment (*p* < 0.001), whereas the proportions of other monosaccharides, especially glucose and mannuronic acid, were concentrated in the solid residue. Also, ash content decreased significantly by 30% (*p* = 0.023). The total carbohydrate content exerted a net increase after pretreatment, but not at a significant level (*p* = 0.086).

### 2.2. Extraction of Polysaccharides from S. latissima by Various Conventional Methods

#### 2.2.1. Three Consecutive Extractions Using Diluted Acid Solution

**Figure 1:**
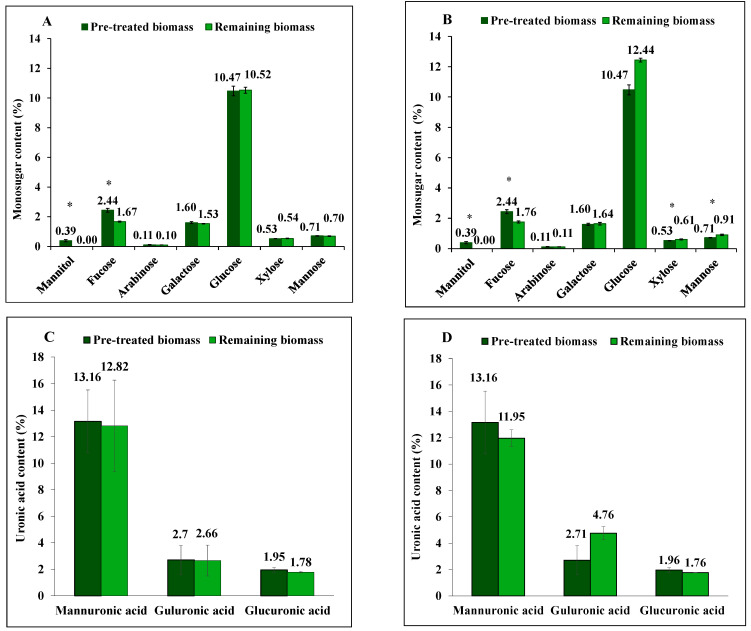
The content of mannitol, neutral monosugars and uronic acids in *S. latissima* biomass before and after 3-step extraction as pre-treated and remaining biomass, respectively, using (**A**,**C**) 0.01 M HCl, and (**B**,**D**) 0.1 M HCl. Data represent the mean ± SD in percentage per dry weight (d.w.) corresponding to three experimental replicates. Asterisk (*) indicates a significant difference (*p* < 0.05) after extraction compared to pretreated biomass (Table 2).

Diluted acid treatment of *S. latissima* managed to extract fucose (the primary monosaccharide in fucoidan) using both HCl solutions, and the yield was calculated based on the difference between initial amount of pre-treated and remaining biomass before and after extraction, respectively, expressed as a percentage of the initial amount (Figure 1A,B). The yield of extracted fucose by 0.01 M HCl was slightly higher (31%, *p* < 0.001) than that obtained by 0.1 M HCl (28%, *p* < 0.001). However, this method proved ineffective for extracting laminarin from the seaweed biomass studied. In the extraction using 0.01 M HCl, the glucose (the primary monosaccharide in laminarin) yield remained unchanged, whereas extraction with 0.1 M HCl resulted in a significantly (*p* < 0.001) higher glucose content in the solid residue compared to the initial content observed prior to extraction.

Furthermore, as illustrated in Figure 1C,D, a 9% reduction in mannuronic acid (from alginate) content following extraction with 0.1 M HCl was observed, however, not at a significant level (*p* = 0.979). In contrast, the content of guluronic acid (from alginate) showed a net increase (*p* = 0.05) in the remaining biomass following extraction with 0.1 M HCl. No significant difference was observed in the concentration of glucuronic acid before and after diluted acid treatment.

#### 2.2.2. Three Consecutive Extractions Using Diluted Alkali Solution

**Figure 2:**
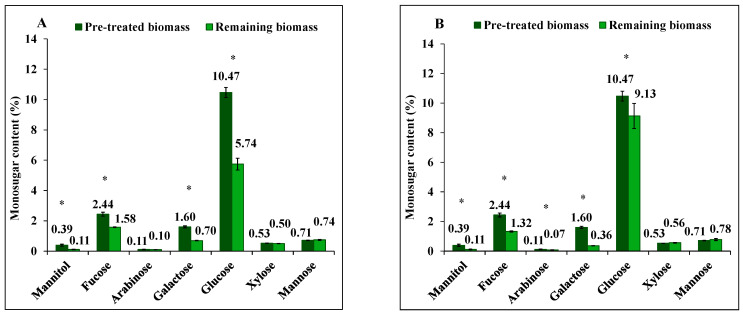

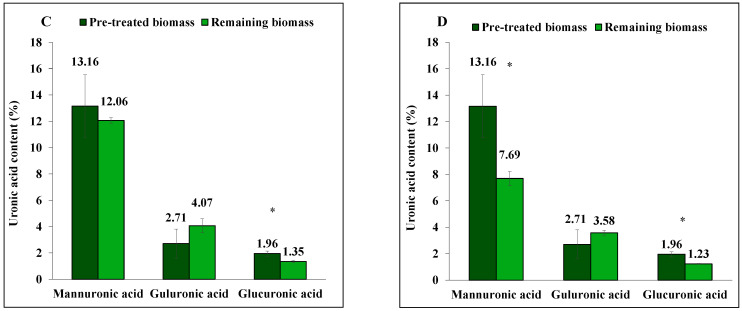
The content of mannitol, neutral monosugars and uronic acids in *S. latissima* biomass before and after 3-step extraction as pre-treated and remaining biomass, respectively, (**A**,**C**) using 0.01 M NaOH, and (**B**,**D**) 0.1 M NaOH. Data represent the mean ± SD in percentage per dry weight (d.w.) corresponding to three experimental replicates. Asterisk (*) indicates a significant difference (*p* < 0.05) after extraction compared to pretreated biomass (Table 2).

Similar to the diluted acid treatment, both diluted alkali solutions also managed to extract fucose (fucoidan) from *S. latissima*, and the yield was calculated based on the difference between initial amount of pre-treated and remaining biomass before and after extraction, respectively, expressed as a percentage of the initial amount (Figure 2A,B). The yield of extracted fucose by 0.1 M NaOH was significantly higher (46%, *p* < 0.001) than that achieved by 0.01 M NaOH (35%, *p* < 0.001). Also, arabinose, galactose, and glucose were extracted using 0.1 M NaOH with yields of 35% (*p* < 0.001), 78% (*p* < 0.001), and 13% (*p* = 0.025), respectively, and from 0.01 M NaOH with yields of 11% (not significant, *p* = 0.630), 56% (*p* < 0.001), and 45% (*p* < 0.001), respectively.

Extraction of mannuronic acid (alginate) differed between 0.01 M and 0.1 M NaOH solutions, yielding 8% (not significant, *p* = 0.993) and 42% (*p* = 0.032), respectively. Guluronic acid (alginate), however, was not extracted with either alkali solution. Instead, it had a net increase in the remaining biomass after extraction. Glucuronic acid extraction yield was 37% (*p* = 0.007) and 24% (*p* < 0.001) under the same conditions.

#### 2.2.3. Hot Water Extraction (Thermal Extraction)

**Figure 3:**
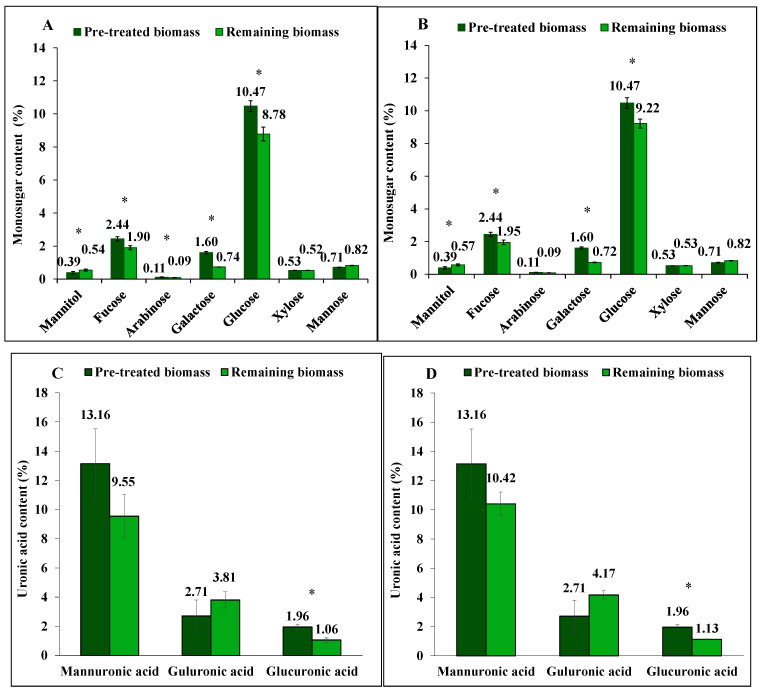
The content of mannitol, neutral monosugars and uronic acids in *S. latissima* biomass before and after hot water extraction as pre-treated and remaining biomass, respectively, for (**A**,**C**) 30 min, and for (**B**,**D**) 60 min. Data represent the mean ± SD in percentage per dry weight (d.w.) corresponding to three experimental replicates. Asterisk (*) indicates a significant difference (*p* < 0.05) after extraction compared to pretreated biomass (Table 2).

The obtained yield of fucose (fucoidan) extracted by hot water was similar at extraction times of 30 min (22%, *p* < 0.001) and 60 min (20%, *p* < 0.001), as seen in Figure 3A,B where the yield was calculated as the percentage of initial amount of pre-treated biomass removed during extraction. Also, arabinose, galactose, and glucose were extracted with yield of 22% (*p* = 0.040), 54% (*p* < 0.001), and 16% (*p* = 0.004), respectively, after 30 min, and 21% (*p* = 0.056), 55% (*p* < 0.001), 12% (*p* = 0.040), respectively, after 60 min.

While not at a significant level, the extraction yield of mannuronic acid (alginate) using hot water was 27% (*p* = 0.254) after 30 min and 21% (*p* = 0.537) after 60 min. Under the same conditions, glucuronic acid yields were 46% (*p* < 0.001) and 42% (*p* < 0.001), respectively. Guluronic acid (alginate) was not extracted by either method, and, similar to the other extraction methods, its proportion increased in the remaining biomass after extraction.

### 2.3. Extraction of Polysaccharides from S. latissima by Two Advanced Techniques

#### 2.3.1. Pressurized Liquid Extraction (PLE)

The Box–Behnken experimental design was used to evaluate the effects of temperature (40, 80, and 120 °C) and extraction time (10, 20, and 30 min) across three levels at a constant pressure of 100 bars. Model fitting plots (Supplementary file: Figure S1) for fucoidan, laminarin, and alginate (polymers of fucose, glucose, and mannuronic/guluronic acids, respectively) showed moderate fits, with R^2^ values of 0.69, 0.72, and 0.72, explaining 69%, 72%, and 72% of the response variability. However, Q^2^ values of 0.27, 0.27, and −0.17 indicated limited to unreliable predictive power, suggesting challenges in generalizing the models, and may need further refinement or more variables.

Coefficient plots (Supplementary file: Figure S1) revealed diverse effects: increased temperature influenced fucoidan and alginate solubilization positively but affected laminarin negatively, while increased time impacted fucoidan and laminarin positively but affected alginate negatively. Quadratic terms showed complex trends: a negative temperature quadratic for fucoidan suggested an optimal peak followed by a decline, whereas positive quadratic terms for temperature and time in alginate and laminarin indicated initial decreases with subsequent increases at higher levels. The interaction term (temperature × time) was negative across all, suggesting that simultaneous increases might lead to degradation or reduced efficiency despite individual positive or negative effects.

**Figure 4:**
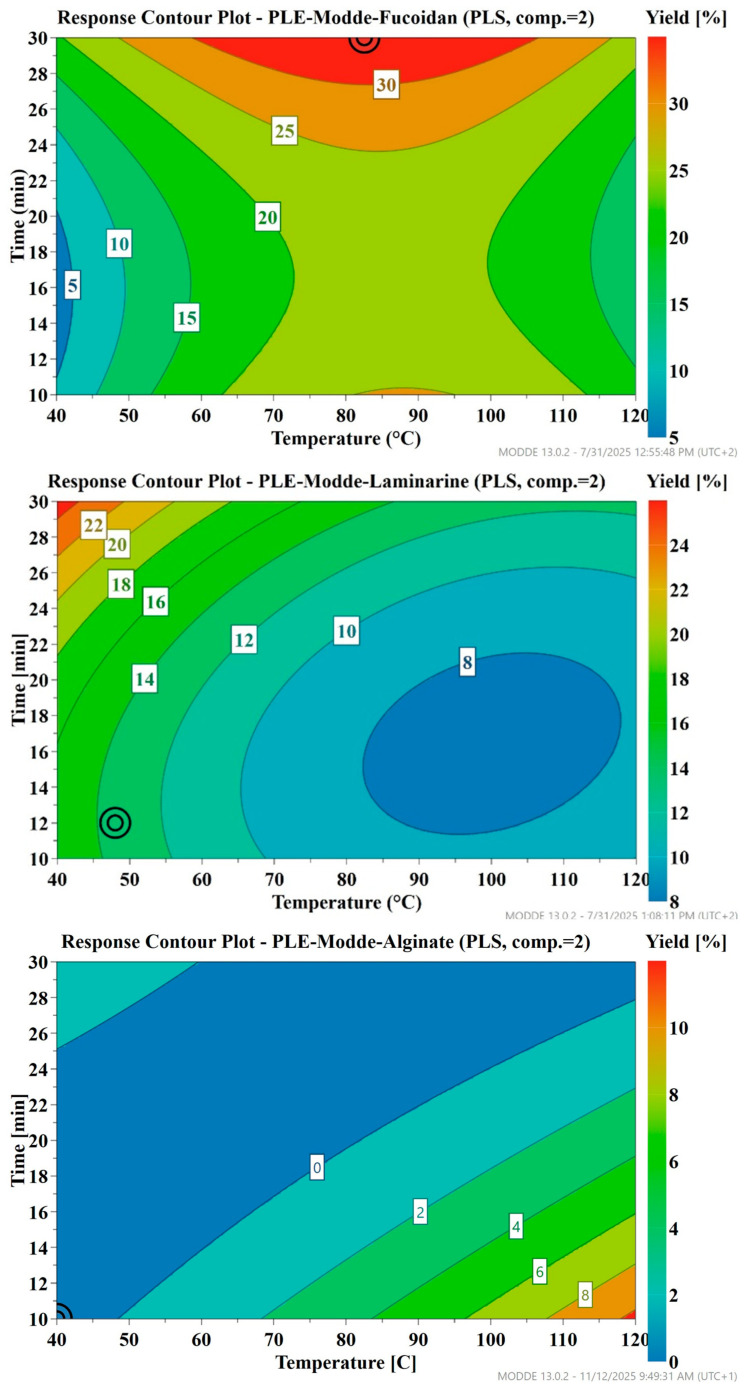
Response Contour plot of the yield of extracted polysaccharides (fucoidan%, laminarin%, and alginate%) as a function of temperature and extraction time, under pressurized hot water extraction at 100 bars. The circled point (⊙) denotes the optimum set point, corresponding to the conditions predicted to give the maximum yield.

Contour plots (Figure 4), based on extraction yields, showed the optimal conditions for isolating the three polysaccharides. Moderate-to-high temperatures and longer times were optimal for enhanced fucoidan solubilization, while higher temperatures with shorter times favored alginate solubilization, and lower temperatures with extended times improved laminarin extraction.

#### 2.3.2. Moderate Electric Field (MEF) Extraction on Solid Residues After Three Consecutive Extractions by Diluted Acid

In this study, the pre-treated samples with ethanol were initially used for electrical-assisted treatment. However, because of the intense conductivity of the brown algae, which is attributed to the high amounts of minerals in *S. latissima*, the electrical methods were not easily applied. Therefore, the solid residues obtained from three consecutive extractions by diluted acid were used instead, since a proportion of the minerals in these samples had been removed, and were hence better suited for the MEF process.

As seen in Table 3, MEF extraction resulted in an increased content of residual neutral monosugars and uronic acids compared to the control, suggesting less effective extraction under given operation conditions (Table 6). Notably, samples 6 and 9 showed a slight decrease in uronic acid content (27.49 ± 4.70%), implying improved extraction, although these samples exhibited significantly higher ash and mineral content (17.48 ± 7.72%). This increase may be due to the extraction of uronic acids, concentrating the minerals in the residue. The remaining content of each neutral monosugar and uronic acid in the solid residue of *S. latissima* after sequential dilute acid and MEF extraction are presented in (Supplementary file: Tables S1 and S2).

### 2.4. Isolation and Characterization of Crude Extracts

Following the extraction of alginate and fucoidan-laminarin mixtures using various conventional methods, the resulting crude extracts were isolated and analyzed for their composition. Due to the limited amount of precipitated crude extract, the composition analysis was carried out in a single replicate.

As shown in Table 4, fucose was the predominant sugar in the crude extracts of fucoidan (fucose) and laminarin (glucose), with the highest proportion observed across all extraction methods except for the extraction with 0.1 M NaOH, where its content was significantly lower (1.8%). In contrast, glucose, which is the main sugar in laminarin, was recovered in very low amount, with the highest recovery being only 4.6% from the 0.01 M NaOH extraction. These results indicate that the isolation of fucoidan was considerably more efficient than that of laminarin due to a higher extraction yield of fucoidan in all methods tested.

Table 5 presents the key components in the crude extract of alginate obtained through all conventional extraction methods. The crude alginate composition is primarily characterized by mannuronic acid, with hot water extraction (particularly at 30 min) proving most effective for its recovery. Fucose content fluctuates, reaching its highest level with mild alkaline extraction (0.01 M NaOH). The use of CaCl_2_ to precipitate alginate also resulted in the co-precipitation of fucoidan, indicating a limitation in selectively isolating alginate. The selection of extraction technique thus significantly impacts the relative proportions of these key components, with thermal methods favoring mannuronic acid.

## 3. Discussion

The ethanol-based pre-treatment effectively reduced extractives such as mannitol and ash while enhancing the neutral monosugar and uronic acid content. The removal of mannitol suggests the potential for selective extraction strategies aimed at obtaining it separately. Additionally, the isolation of phenolic compounds has previously been demonstrated under the same conditions [14].

The findings of Ale et al. (2011) showed that diluted acid extraction effectively recovered fucoidan [15]. Although their analysis was based on colorimetric chemical methods applied to different brown seaweed species, the results were consistent with our observation that fucoidan extraction was enhanced using 0.01 M and 0.1 M HCl [15,16]. However, laminarin was not solubilized under these conditions, and while acid-based laminarin extraction has been studied, yields were not reported [5,17]. Dilute acid extraction yielded 10% of alginate, which is lower than the 28.81% reported by Fawzy et al. (2021) using brown seaweed *Sargassum latifolium* [18]. However, Fawzy et al. (2021) employed acid depolymerization method for determination of alginate yield, and their results indicated that lower acid concentrations combined with higher temperatures led to an increase in extraction of mannuronic acid rather than guluronic acid [18].

Alkaline extraction using both 0.1 M and 0.01 M NaOH enhanced the solubilization of fucoidan-associated sugars, particularly fucose, with yields of 46% and 35%, respectively. The higher yield obtained with 0.1 M NaOH suggested a more pronounced disruption of the algal cell matrix, promoting fucoidan release when compared to extraction under more diluted acidic conditions. However, the lower relative proportion of fucose in the crude extract from the 0.1 M NaOH treatment may indicate partial degradation of fucoidan under stronger alkaline conditions [19]. In contrast, the extraction of glucose, primarily associated with laminarin, remained limited under the 0.1 M NaOH extraction (13%), while a considerably higher glucose yield (45%) was achieved with 0.01 M NaOH, suggesting that milder alkaline conditions are favorable for laminarin solubilization compared to acid-based extraction. Lim et al. (2017) [20] previously described the effectiveness of alkaline extraction for both fucoidan and laminarin recovery. The alkali extraction yielded 8% (0.01 M NaOH) and 42% (0.1 M NaOH) of alginate, which corresponded to the presence of mannuronic acid. Most literature reports alginate extraction under alkaline conditions following an initial acid treatment, with the highest yield (up to 90% from *Ascophyllum nodosum* typically expressed as the weight of the precipitated crude alginate [21].

Hot water extraction or thermal extraction (121 °C, 1.03 bar) resulted in a moderate recovery of fucose (20–22%), while glucose recovery in the extract remained minimal (< 4%). These results indicate that fucoidan can be extracted thermally without the use of chemical reagents. The yield of extracted fucoidan was higher than that reported by Moreira et al. (2023) [4], who achieved a yield of 11.4 mol% (10.54 wt%) from *Saccharina latissima* using GC-FID analysis. In contrast, high temperature alone was not sufficient for effective laminarin recovery. The results were different from those of Moreira et al. (2023) [4], in which a significantly higher glucose yield of 35.3 mol% (34.9 wt%) was reported. This discrepancy may be attributed to the lower extraction temperature (90 °C) used in their study, which might have been favorable for laminarin solubilization. Interestingly, this method was most effective for alginate extraction, yielding 27% of mannuronic acid. which is among the highest yields reported to date [10].

Notably, in all extraction processes, predominantly mannuronic acid-rich fractions were solubilized, while guluronic acid-rich fractions remained in the solid residues. This behavior could be attributed to the formation of strong Ca^2+^-mediated “egg-box” junctions between G-blocks within the brown-algal cell wall. These Ca-alginate networks exhibit low solubility and are more resistant to conversion into Na-alginate under extraction conditions [10,22]. Consequently, the more soluble M-rich fractions were extracted, leaving behind a G-enriched, Ca-bound residue.

The extraction of functional ingredients from natural sources using advanced and eco-friendly technologies is essential, as these approaches address the significant environmental limitations associated with conventional extraction methods [23]. To achieve higher yields of the target analyte, various parameters must be optimized.

Pressurized Liquid Extraction (PLE) is an environmentally friendly technique that enables the efficient recovery of compounds from solid matrices under elevated temperatures (50–200 °C) and pressures (3.5–20 MPa). It has shown excellent performance in isolating a variety of bioactive compounds from different natural sources while significantly reducing solvent consumption and extraction time. Compared with conventional extraction methods, PLE provides markedly shorter extraction durations typically 5–20 min and higher yields. The technique supports the use of various solvents, including green alternatives such as water, making it highly suitable for sustainable processing. Therefore, PLE holds strong potential for applications within the food industry [24]. Previous study has demonstrated extraction of phlorotannins from *S. latissima*; however, its capability for polysaccharide extraction from this biomass remained unexplored [11].

In this study, Pressurized Liquid Extraction (PLE) at a constant pressure of 100 bars was employed to assess how variations in temperature and extraction time influence polysaccharide recovery under initial experimental conditions. At this stage, the primary goal of using Pressurized Liquid Extraction (PLE) was to investigate how the technique performs for extracting seaweed polysaccharides and to establish its baseline extraction behavior. The parameters were therefore selected to explore the general response of PLE under controlled conditions, rather than to directly replicate or optimize based on the conventional extraction results.

The results indicated that the three target polymers required different conditions for optimized isolation. Therefore, the optimized conditions suggested by the model were not implemented, as they could not be applied simultaneously for all polysaccharides and were supported by low Q^2^ values (0.27 for fucoidan and laminarin, −0.17 for alginate), indicating limited predictive reliability. Further experiments are needed to validate these predictions and strengthen the model.

Moderate Electric Field (MEF) extraction is a promising, green, and energy-efficient alternative to conventional methods. By inducing electroporation and enhancing cell membrane permeability, MEF enables faster, milder, and more selective extraction of bioactive compounds and biopolymers from various biological materials, while preserving their structural integrity and functional properties [25]. Moderate electric field (MEF) extraction has predominantly been used for recovering pigments, polyphenols, and proteins from seaweed, whereas its application for polysaccharide extraction remains relatively limited [26], which impedes comparisons with the literature. A key challenge encountered in this study was the naturally high electrical conductivity of *Saccharina latissima*, largely attributed to its elevated ash content. This observation is consistent with findings from previous studies [27], which highlight that the high ionic content of seaweed can hinder the effective implementation of electric-field-based extraction methods. To circumvent this challenge, MEF was applied to acid-washed residues, which had reduced ionic concentrations. However, residual conductivity continued to influence extraction efficiency. Although MEF did not markedly enhance the overall polysaccharide extraction yield, selective improvements were noted under certain conditions, particularly in samples 6 and 9, which exhibited the lowest residual uronic acid content. These findings suggest that, with optimized process parameters, MEF could facilitate mechanisms such as cell wall loosening or electro-permeabilization, thereby promoting uronic acid release. However, more research is needed to develop and optimize this process.

## 4. Materials and Methods

### 4.1. Materials

Biomass of *Saccharina latissima* (L.) C.E.Lane, C.Mayes, Druehl & G.W.Saunders 2006 in powder form (Batch: S-B-1707-D-1-170914-5-L) was received from Oceans Rainforest (Kaldbak, Faroe Islands). This sample was harvested in May 2017 and dried on site, and the particle size was around 0.5 mm.

All chemicals were supplied from Merck (Sigma-Aldrich, Stockholm, Sweden) unless otherwise stated.

### 4.2. Methods

#### 4.2.1. Pre-Treatment of *S. latissima*

Thirty milliliters of aqueous ethanol solution (80%, *v/v*) was added to 3 g of seaweed in 50 mL Falcon tubes, which were incubated at 25 °C for 20 h and subsequently at 65 °C and 150 rpm for 5 h in a shaker incubator (Ecotron, Infors, Basel-Landschaft, Switzerland). Then, the samples were centrifuged at 3893× *g* for 10 min (Sigma 3-16PK). The supernatants were collected, and another 30 mL of fresh aqueous ethanol solution (80%, *v/v*) was added to the seaweed residues and placed in the shaker for 5 h at 65 °C with constant shaking at 150 rpm. Then, the samples were centrifuged at 3893× *g* for 10 min, and the supernatants were collected. The solid residue containing pre-treated seaweed was washed with distilled water, centrifuged at 3893× *g* for 10 min, air-dried with open lid, and stored at room temperature for further analysis. A parallel experiment was performed where the pre-treated seaweed was washed with distilled water and used for further experiments without air-drying.

#### 4.2.2. Extraction of Polysaccharides from Pre-Treated *S. latissima* by Various Conventional Methods

##### Three Consecutive Extractions Using Diluted Acid Solution

Diluted HCl solutions with concentrations of 0.01 M and 0.1 M were prepared, and 30 mL of each solution was added to 3 g of pre-treated seaweeds in 50 mL Falcon tubes. The Falcon tubes were placed in a shaker incubator (Ecotron, Infors Basel-Landschaft, Switzerland) at 65 °C for 3 h and centrifuged at 3893× *g* for 10 min, and then the supernatants were collected. The extraction procedure was repeated twice under the same conditions for both acid concentrations. After that, the solid residues from each extraction step were washed 3 times with distilled water, centrifuged at 3893× *g* for 10 min, open air-dried, and stored at room temperature for further analysis. The collected supernatants of the repeated extraction experiments for each solution (0.01 M or 0.1 M HCl, separately) were pooled and kept in a freezer (−20 °C) for further experiments.

##### Three Consecutive Extractions Using Diluted Alkali Solution

Diluted NaOH solutions with concentrations of 0.01 M and 0.1 M were prepared, and 30 mL of each solution was added to 3 g of pre-treated seaweeds in 50 mL Falcon tubes. The Falcon tubes were placed in a shaker incubator at 65 °C for 3 h, centrifuged at 3893× *g* for 10 min, whereafter the supernatants were collected. The extraction procedure was repeated twice under the same conditions for both alkali concentrations. After that, the solid residue of each experiment was washed 3 times with distilled water, centrifuged at 3893× *g* for 10 min, open air-dried, and stored at room temperature for further analysis. The collected supernatants of the repeated extraction steps for each solution (0.01 M or 0.1 M NaOH, separately) were pooled and kept in a freezer (−20 °C) for further experiments.

##### Hot Water Extraction (Thermal Extraction)

Thirty milliliters of distilled water was added to 3 g of pre-treated seaweed in 100 mL glass bottles with screw caps. The bottles were placed in an autoclave at 121 °C (≈1.03 bar) for 30 min or 60 min, separately. After autoclavation, the mixtures were transferred to 50 mL Falcon tubes and centrifuged at 3893× *g* for 10 min. The supernatants were collected and kept in a freezer (−20 °C) for further experiments. The solid residues from each extraction were open air-dried and stored at room temperature for further analysis.

#### 4.2.3. Extraction of Polysaccharides from *S. Latissima* by Two Advance Techniques

##### Pressurized Liquid Extraction (PLE) on Untreated Biomass

Pressurized Liquid Extraction (PLE) was performed using a Dionex ASE-350 system (Thermo Fisher, Germering, Germany). For each extraction, 1 g of untreated *S. latissima* was weighed and loaded into a 10 mL stainless-steel extraction vessel equipped with 0.45 µm metallic filters at both the inlet and outlet. Extractions were conducted in static mode, and following each run, the vessel was flushed with fresh solvent equivalent to 60% of its volume. The remaining contents were then purged with nitrogen gas for 90 s.

In this study, pressurized hot water served as the extraction solvent at a constant pressure of 100 bar. To optimize the extraction conditions, a Box–Behnken experimental design with three center points was constructed using MODDE^®^ 10.1 (Sartorius Stedim Data Analytics AB, Umeå, Sweden). The effects of extraction temperature (T; 40–120 °C) and extraction time (t; 10–30 min) on polysaccharide yield were evaluated. Partial Least Squares (PLS) was applied to develop the predictive model and generate the response surface. Model performance was assessed using R^2^ (model fit) and Q^2^ (predictive ability), along with graphs plotting the predicted versus observed outcomes, and coefficients.

##### Moderate Electric Field (MEF) Extraction on Solid Residues After Sequential Diluted Acid Extraction

The solid residue obtained from the diluted acid extraction described in section Three Consecutive Extractions Using Diluted Acid Solution, was used as the starting material (control) for the MEF extraction method. Samples (0.1 g) were placed in an electroporation chamber with a 0.5 cm gap between the electrodes. The chamber was filled with a 50 mM NaCl solution, with an electrical conductivity of 130 μS/cm, ensuring complete coverage of both the electrodes and the samples. The system was then connected to an AC power source (3000 VA, BK Precision Corporation, Yorba Lindam, CA, USA). The temperature increase during MEF extraction remained below 2 °C, enforcing minimal thermal impact.

A total of 11 experimental runs were designed using MODDE^®^ 12.1 Design of Experiments software, as shown in Table 6. Four variables, X_1_ (electric field intensity), X_2_ (frequency), X_3_ (extraction time), and X_4_ (seaweed:water ratio), were tested at different levels. The extraction yields of fucoidan, alginate, laminarin, and ash were recorded as response variables. Changes in temperature (T) and electrical resistance (R) were recorded during and following MEF extraction (Supplementary file: Table S3).

**Table 6:** MEF extraction conditions generated by MODDE^®^ 12.1 Design of Experiments software for acid-extracted samples as starting materials (control).

Sample	X_1_ (V/cm)	X_2_ (Hz)	X_3_ (min)	X_4_ (Seaweed:Water)
1	30	1200	2	1:10
2	50	100	2	1:20
3	70	1200	5	1:20
4	30	100	5	1:10
5	50	1200	10	1:20
6	70	100	10	1:10
7	30	100	15	1:20
8	50	1200	15	1:10
9	70	100	10	1:10
10	50	1200	10	1:20
11	30	100	5	1:10

#### 4.2.4. Isolation of Extracted Polysaccharides

An aqueous solution of CaCl_2_ (2% *w/v*) was prepared and added to the supernatants obtained from the different extraction methods at a ratio of (1:1) in glass bottles with screw caps. Then the solutions were mixed and kept in the fridge at (4 °C) overnight to isolate the alginate from the supernatants. After that, the mixtures were centrifuged at 3893× *g* for 10 min (Sigma 3-16PK), the alginate-free supernatants were transferred to new glass bottles with screw caps, and the resulting precipitated crude alginates were freeze-dried in pre-weighed Falcon tubes to be used for further analysis. The alginate-free supernatants were used for the isolation of a mixture of laminarin and fucoidan. Ethanol solution (99.9%) was added to alginate-free supernatants at a ratio of 4:1 (ethanol:supernatant). The solutions were mixed and kept in fridge at 4 °C overnight. After that, the mixtures were centrifuged at 3893× *g* for 10 min, the supernatants were discarded, and the resulting crude mixture of laminarin and fucoidan precipitate was freeze-dried in pre-weighed Falcon tubes to be used for further analysis.

### 4.3. Analytical Procedures

#### 4.3.1. Ash Content

The ash content was determined by the method described by Van Wychen et al. (2013) [28]. Four crucibles were preconditioned in an incinerator (Nabertherm, Lilienthal, Germany) at 560 °C for 12 h to remove any contaminants. After that, 0.1 g of sample was added to the crucibles and then they were placed in an incinerator at 550 °C for 12 h. After being transferred to a desiccator and allowed to cool to room temperature, the ash content was determined gravimetrically.

#### 4.3.2. Total Carbohydrates Composition Determination

Determination of total carbohydrates of *S. latissima* was performed using a two-step sulfuric acid hydrolysis treatment described by Sluiter et al. (2008) [29]. Initially, 3 mL of sulfuric acid 72% (*w*/*w*) was added to 0.3 g of seaweed powder in 200 mL glass bottles with screw caps, and incubated (Electron, Infors, Basel-Landschaft, Switzerland) at 30 °C and 150 rpm with constant shaking for 60 min. Then, the samples were diluted to a sulfuric acid concentration of 4% (*w*/*w*) using ultrapure water (Milli-Q grade), and were autoclaved at 121 °C for 1 h. After that, the hydrolysate was cooled down to room temperature and centrifuged at 3893× *g* for 10 min.

The supernatant from the hydrolysate was neutralized by 0.1 M barium hydroxide, and centrifugated at 3893× *g* for 5 min. To prepare for analysis, the hydrolysates were diluted properly and filtered through 0.2 µm polypropylene syringe filters. Analysis was performed using a high-performance anion exchange chromatography system equipped with a pulsed amperometric detector (HPAEC-PAD) (Thermo Fisher Scientific, Sunnyvale, CA, USA), and a Dionex CarboPac PA-20 analytical column (150 × 3 mm, 6 µm) with guard column (30 × 3 mm) (Thermo Fisher Scientific, Waltham, DC, USA) used to separate sugars. Separation of monomeric sugars and the sugar alcohol mannitol was performed under isocratic conditions at a flow rate of 0.5 mL/min for 30 min using an eluent mixture of 62.5% (A) ultrapure water, and 37.5% (B) 2 mM sodium hydroxide. The standards used were mannitol, fucose, arabinose, galactose, glucose, xylose, and mannose.

Separation and analysis of uronic acids were also performed under isocratic conditions at a flow rate of 0.5 mL/min for 30 min, with column and compartment temperatures maintained at 30 °C. Uronic acids were eluted by 55% (A) ultrapure water, 15% (B) 1 M sodium acetate, and 30% (C) 200 mM sodium hydroxide. The standards used were glucuronic acid, guluronic acid, and mannuronic acid.

#### 4.3.3. Determination of Sulphate Content in Fucoidan

Quantitative analysis of the sulphate content of extracted crude fucoidan was performed by the method described by Soleimani et al. (2012) [30] with modifications. A UHPLC system (Ultimate-3000 RSLC, Dionex) (Thermo Fisher Scientific, Germering, Germany), connected to a C18 column (Gemini-NX, 100 × 2 mm, 3 μm particle size, 110 Å, Phenomenex) (Phenomenex Inc., Torrance, CA, USA), was used. The mobile phase consisted of aqueous methanol 30% *v*/*v*) with 0.08 mM hexadecyltrimethylammonium bromide (HTAB). Separation was performed by injection of 20 μL of the sample, followed by elution under isocratic conditions at a flow rate of 0.3 mL/min, and a column temperature maintained at 35 °C. Eluted sulphate ions were detected at 206 nm using a UV-Vis detector (Ultimate 3000 RS, Dionex). Standard sulphate solutions were prepared at a range of 1–20 mg/L from a commercial aqueous sulphate standard solution with a concentration of 800 mg/L, and filtered using 0.2 μm syringe filters in HPLC vials before analysis.

#### 4.3.4. Statistical Analysis

Extraction yields refer to the amount of alginate, fucoidan and laminarin content solubilized from the biomass, and are calculated based on the monosugar content, i.e., mannuronic acid, fucose, and glucose. For each extraction method, yields were calculated as the difference between initial (pretreated biomass) and remaining biomass after extraction, expressed as a percentage of the initial amount, according to the equation below:

$$Extraction\;yield\;\left(\%\right)=\frac{monosugar\;content\;in\;pretreated\;biomass\;\left(\%\right)-monosugar\;content\;in\;remaining\;biomass(\%)}{monosugar\;content\;in\;pretreated\;biomass\;(\%)}\times 100$$

Descriptive statistics were calculated for all data when feasible in Microsoft Excel (Version 2509) and presented as mean values with standard deviation. Inferential statistics were performed to investigate potential differences between various treatments by one-way ANOVA with Tukey’s post hoc test using the software jamovi (Version 2.6.44, Australia).

## 5. Conclusions

Various extraction methods were applied to recover polysaccharides from *S. latissima*, demonstrating the potential of a cascading extraction process in which different polysaccharide fractions can be sequentially obtained. Fucoidan was effectively extracted using either diluted HCl or 0.1 M NaOH, while a mixture of fucoidan and laminarin was obtained with 0.01 M NaOH. Hot water extraction for 30 min yielded a mixture rich in alginate, aligning with PLE results that identified high temperature and short extraction time as optimal for alginate recovery. In contrast, MEF did not significantly enhance polysaccharide extraction in this study, likely due to the high ionic content of *S. latissima*, although limited effects were observed under specific conditions.

Although the current fractions still contain some impurities and partial overlap between polysaccharides, further optimization of the downstream purification steps is needed to improve the selectivity and yield of the targeted polysaccharides, leading to higher-purity fractions, paving the way for more efficient and sustainable seaweed biorefinery strategies.

## Figures and Tables

**Table 1:** Composition of untreated *S. latissima* biomass. Data represent the mean ± SD in percentage per dry weight (d.w.) corresponding to three experimental replicates.

Total Carbohydrate (%)	Ash (%)	Monosugars (%)						
27.4 ± 4.05	49.47 ± 9.37	Mannitol	Fucose	Arabinose	Galactose	Glucose	Xylose	Mannose
		7.14 ± 0.45	2.08 ± 0.18	0.08 ± 0.004	1.06 ± 0.13	6.15 ± 0.63	0.34 ± 0.03	0.50 ± 0.03
		Uronic acids (%)						
		Mannuronic acid			Guluronic acid		Glucuronic acid	
		7.12 ± 1.97			1.94 ± 1.11		0.97 ± 0.05	

**Table 2:** Composition of pre-treated *S. latissima* biomass with aqueous ethanol solution (80% *v*/*v*). Data represent the mean ± SD in percentage per dry weight (d.w.) corresponding to three experimental replicates. Asterisk (*) indicates a significant difference (*p* < 0.05) after pretreatment in comparison to untreated biomass in Table 1.

Total Carbohydrate (%)	Ash (%)	Monosugars (%)						
33.8 ± 2.75	19.35 ± 1.2	Mannitol	Fucose	Arabinose	Galactose	Glucose	Xylose	Mannose
		0.39 ± 0.07 *	2.43 ± 0.38 *	0.11 ± 0.01 *	1.60 ± 0.06 *	10.47 ± 0.32 *	0.39 ± 0.07 *	2.43 ± 0.38 *
		Uronic acids (%)						
		Mannuronic acid			Guluronic acid		Glucuronic acid	
		13.16 ± 2.37 *			2.70 ± 1.10		1.95 ± 0.19 *	

**Table 3:** Effect of MEF extraction and its operating conditions (Table 6) on the yield of neutral monosugars and uronic acids, compared to the acid-extracted residue used as the control in *S. latissima.* Data represent the mean ± SD in percentage per dry weight (d.w.) corresponding to two experimental replicates.

Experiment	Total Remaining Neutral Monosugars in Residue (%)	Total Remaining Uronic Acids in Residue (%)	Ash (%)
Control	10.68	30.49	10.36 ± 0.22
1	13.01	39.05	11.11
2	12	34.57	10.86
3	12.47	39.44	10.55
4 and 11	13.14 ± 1.00	33.55 ± 1.56	10.12 ± 0.31
5 and 10	13.285 ± 0.76	33.46 ± 1.42	10.565 ± 0.07
6 and 9	11.865 ± 2.62	27.49 ± 4.70	17.475 ± 7.72
7	13.2	34.98	11.02
8	12.5	32.08	10.83

**Table 4:** Composition of key components in the crude liquid extract of fucoidan and laminarin obtained through conventional extraction of *Saccharina latissima* biomass.

Extraction Method	Composition (%)			
	Fucose	Glucose	Mannuronic Acid	Sulphate
Extraction using 0.01 M HCl	15.5	2	4.1	0.72
Extraction using 0.01 M NaOH	14.4	4.6	4.7	0.15
Extraction using 0.1 M NaOH	1.8	4.15	0.24	0.06
Extraction using hot water (30 min)	14.8	3.9	37.6	0.12
Extraction using hot water (60 min)	15	1.65	6.3	0.10

**Table 5:** Composition of key components in the crude extract of alginate obtained through conventional extraction of *Saccharina latissima* biomass.

Extraction Method	Composition (%)	
	Fucose	Mannuronic Acid
Extraction using 0.01 M HCl	6.83	3.22
Extraction using 0.01 M NaOH	8.82	5.16
Extraction using 0.1 M NaOH	0.0	7.33
Extraction using hot water (30 min)	1.44	25.7
Extraction using hot water (60 min)	1.12	22

## Data Availability

The data presented in this study are available on request from the corresponding author.

## Supplementary Materials

The following supporting information can be downloaded at: https://www.mdpi.com/article/10.3390/md23110435/s1, Figure S1. Validation plots for the PLE show the model fitting (R2) and prediction power (Q) and coefficient plots for fucoidan, laminarin, and alginate. The coefficient plots show the direct and interaction effects of the investigated parameters on the solubility of the corresponding polysaccharides. Table S1. Remaining monosugars (% seaweed biomass) in the solid residue of *S. latissima* after combined diluted acid and MEF extraction. Table S2. Remaining uronic acids (% seaweed biomass) in the solid residue of *S. latissima* after combined diluted acid and MEF extraction. Table S3. Changes in temperature (T) and electrical resistance (R) observed during and after MEF extraction.
