# Supplementary material for: Impact of Individual Process Parameters on Extraction of Polysaccharides from Saccharina latissima
Source: Mar Drugs. 2025 Nov 13;23(11):435. doi: 10.3390/md23110435 (PMC12654208; doi:10.3390/md23110435)
Supplement: Supplementary file 1 [file marinedrugs-23-00435-s001.zip › marinedrugs-3942240-supplementary.pdf]

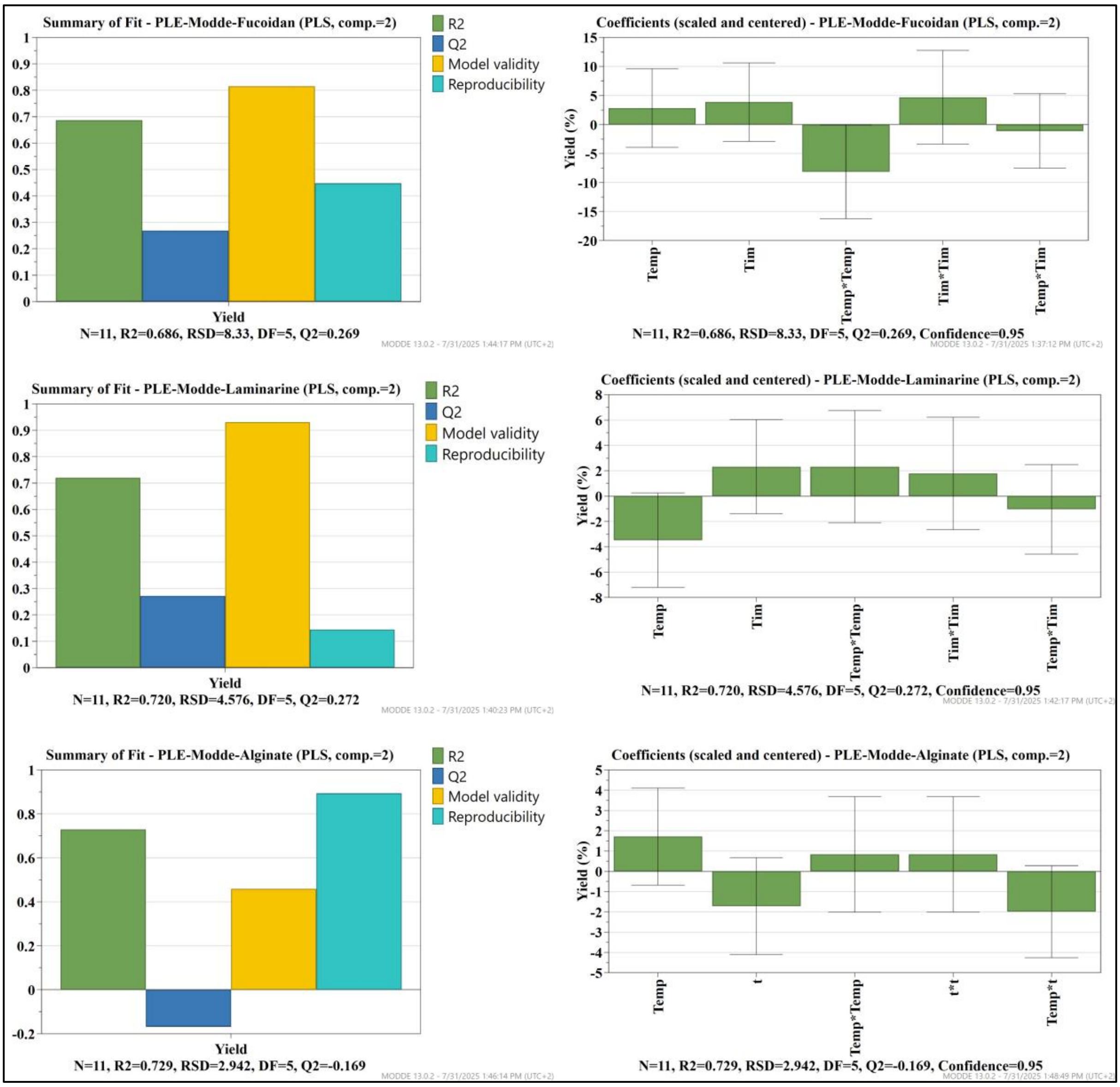

Supplementary Figure S1. Validation plots for the PLE show the model fitting (R2) and prediction power (Q) and coefficient plots for fucoidan, laminarin, and alginate. The coefficient plots show the direct and interaction effects of the investigated parameters on the solubility of the corresponding polysaccharides.

Table S1. Remaining monosugars (% seaweed biomass) in the solid residue of *S. latissima* after combined diluted acid and MEF extraction**.**

Number of experiments	Fucose	Arabinose	Galactose	Glucose	Xylose	Mannose
Control	1.17±0.02	0.07±0.00	0.85±0.01	7.47±0.16	0.47±0.02	0.65±0.00
1	1.34	0.09	0.99	9.18	0.55	0.86
2	1.50	0.08	1.00	8.39	0.48	0.55
3	1.48	0.09	1.00	8.54	0.59	0.77
4 and 11	1.48±0.02	0.09±0.00	0.99±0.05	8.98±0.78	0.58±0.01	0.84±0.03
5 and 10	1.50±0.06	0.09±0.01	1.00±0.03	8.98±0.28	0.51±0.08	0.71±0.12
6 and 9	1.32±0.26	0.08±0.01	0.88±0.23	7.77±1.58	0.52±0.17	0.67±0.23
7	1.34	0.09	0.95	9.36	0.58	0.88
8	1.32	0.09	0.92	8.91	0.53	0.73

Table S2. Remaining uronic acids (% seaweed biomass) in the solid residue of *S. latissima* after combined diluted acid and MEF extraction.

Experiment	Guluronic acid	Glucuronic acid	Mannuronic acid
Control	9.24±0.07	4.16±0.30	17.09±0.40
1	11.08	5.12	22.85
2	9.98	5.15	19.44
3	11.18	5.22	23.04
4 and 11	9.93±0.15	4.54±0.15	19.08±0.81
5 and 10	10.07±0.04	4.44±0.08	18.95±0.89
6 and 9	8.83±0.66	3.67±0.50	14.99±2.17
7	10.37	4.53	20.08
8	9.34	4.41	18.33

Table S3. Changes in temperature (T) and electrical resistance (R) observed during and after MEF extraction.

Number of experiments	T0 (℃) (At the start)	T1(℃) (At the end)	R0 (Ω) (At the start)	R1 (Ω) (At the end)
Control	-	-	-	-
1	22.8	23.2	434	422
2	22.9	22.5	319	291
3	22.5	30.7	229	170
4	24.3	23.9	386	374
5	22.7	28.4	236	195
6	24	26.5	379	380
7	22.8	23.2	288	236
8	22.5	26.9	335	349
9	22.8	27.2	353	265
10	23.2	29.8	253	173
11	24	24.3	352	359
